# Faster than light (microscopy): superiority of digital pathology over microscopy for assessment of immunohistochemistry

**DOI:** 10.1136/jclinpath-2021-207961

**Published:** 2022-01-17

**Authors:** Emily Clarke, Daniel Doherty, Rebecca Randell, Jonathan Grek, Rhys Thomas, Roy A Ruddle, Darren Treanor

**Affiliations:** 1 Division of Pathology and Data Analytics, University of Leeds, Leeds, UK; 2 Department of Histopathology, Leeds Teaching Hospitals NHS Trust, Leeds, UK; 3 Faculty of Health Studies, University of Bradford, Bradford, UK; 4 Wolfson Centre for Applied Health Research, Bradford, UK; 5 Northern Ontario School of Medicine, Thunder Bay, Ontario, Canada; 6 School of Computing and Leeds Institute of Data Analytics, University of Leeds, Leeds, UK

**Keywords:** immunohistochemistry, diagnostic techniques and procedures, pathology, surgical, computer systems

## Abstract

**Aims:**

Digital pathology offers the potential for significant benefits in diagnostic pathology, but currently the efficiency of slide viewing is a barrier to adoption. We hypothesised that presenting digital slides for simultaneous viewing of multiple sections of tissue for comparison, as in those with immunohistochemical panels, would allow pathologists to review cases more quickly.

**Methods:**

Novel software was developed to view synchronised parallel tissue sections on a digital pathology workstation. Sixteen histopathologists reviewed three liver biopsy cases including an immunohistochemical panel using the digital microscope, and three different liver biopsy cases including an immunohistochemical panel using the light microscope. The order of cases and interface was fully counterbalanced. Time to diagnosis was recorded and mean times are presented as data approximated to a normalised distribution.

**Results:**

Mean time to diagnosis was 4 min 3 s using the digital microscope and 5 min 24 s using the light microscope, saving 1 min 21 s (95% CI 16 s to 2 min 26 s; p=0.02), using the digital microscope. Overall normalised mean time to diagnosis was 85% on the digital pathology workstation compared with 115% on the microscope, a relative reduction of 26%.

**Conclusions:**

With appropriate interface design, it is quicker to review immunohistochemical slides using a digital microscope than the conventional light microscope, without incurring any major diagnostic errors. As digital pathology becomes more integrated with routine clinical workflow and pathologists increase their experience of the technology, it is anticipated that other tasks will also become more time-efficient.

## Introduction

Over the past few years, digital pathology has been deployed for primary diagnosis in a few flagship projects around the world. Our institution now routinely digitally scans 100%^1^ of the cases coming through the department and it has become clear that these systems are able to offer numerous benefits to pathology departments such as improved workflow, reduced impact of human error and increased efficiency in diagnostic work.^2^ However, there is still insufficient research evaluating the likely advantages of digital pathology,^3^ which, in combination with poorly integrated software and high initial cost outlay, has hindered uptake^4^ particularly in smaller institutions.

Of late, much of the research has been concerned with diagnostic accuracy as the introduction of a new technology must not compromise patient safety. Many single papers and a systematic review^5^ have confirmed non-inferiority of the digital microscope when compared with the light microscope. Additionally, two systems have been approved by the US Food and Drug Administration for primary diagnosis.^6 7^ However, accuracy is not the only aspect for concern—time taken to reach a diagnosis is also of great importance. The longer it takes to reach a diagnosis, the more effort is required by the pathologist, resulting in a reduction in their productivity. This is critical given the increasing demand for pathology services alongside a dramatic increase in retirement rate of pathologists within the UK,^8^ with only 3% of pathology departments being fully staffed.^9^

Preliminary work from our group showed that early whole slide imaging (WSI) viewers were 60% slower than the microscope,^10^ which posed a major barrier to adoption. This work led to the development and design of WSI software to focus on the need for fast viewing—the Leeds Virtual Microscope.^11 12^ We have shown that a digital microscope could be as quick as a light microscope for diagnostic purposes but was not faster, both using a wall-sized display and an 8-megapixel desktop setup, applied to a variety of diagnostic tasks.^2^

A more recent development of this software though allows for simultaneous viewing of multiple sections of tissue for comparison, something that is simply not possible with light microscopy. With as little as 61.9% of a pathologist’s time spent viewing an image when at the microscope,^4^ the rest of the time being consumed with manual processes such as removing slides from slide trays, adjusting slides on the microscope stage and dictating the report, we anticipate that viewing multiple slides side by side will be of significant time benefit to pathologists. It will be particularly beneficial in large resection cases, or cases where there are multiple immunohistochemical-stained slides (approximately 13% of cases within our institution).

We therefore designed an experiment to compare the time with diagnosis using a digital system with the microscope. The digital system minimises the effort to manually switch between separate slides, instead offering the user a one-touch method of reviewing the case. It is hypothesised that the use of an appropriately designed digital pathology workstation can offer a reduction in time to reach a diagnosis without compromising diagnostic confidence.

## Materials and methods

A purposive sampling strategy was employed to recruit 16 participants from within our institution: 8 senior trainee histopathologists and 8 consultant histopathologists from a range of subspecialist fields (not including liver pathology). Pathologists were recruited in person and those included were those who were approached and were willing to be involved. This study took place prior to the digitisation of our department resulting in a wide range of experience of using a digital microscope, from very little to many days’ cumulative experience.

All participants were asked to view three cases using the digital microscope, and three different cases using the light microscope. The order of cases and interface was fully counterbalanced.

All cases were liver needle core biopsies of tumours, with clinical details available in table 1. Liver biopsy cases were chosen since many tumours are metastatic and therefore require a large panel of immunohistochemical stains to identify the location of the primary tumour. The cases were selected from archives at our institution and reviewed by a consultant histopathologist (DT). Three cases were designated as ‘set A’ and the other three as ‘set B’. Equal numbers of slides were included in each set. Details of these cases can be found in online supplemental table 1.

10.1136/jclinpath-2021-207961.supp2 Supplementary data

**Table 1:** A summary of the main results

	Mean time to diagnosis/case, (m=minutes; s=seconds)			Normalised time to diagnosis per case, %		
	Light microscope	Digital microscope	Difference	Light microscope	Digital microscope	Difference
Overall	5 m 24 s	4 m 3 s	1 m 21 s (95% CI 0 m 16 s to 2 m 26 s), p=0.02	115	85	30 (95% CI 15 to 45), p=0.0006
Trainees only	5 m 25 s	3 m 31 s	1 m 54 s (95% CI 0 m 37 s to 3 m 11 s), p=0.007	116	74	42 (95% CI 14 to 70), p=0.006
Consultants only	5 m 24 s	4 m 35 s	0 m 48 s (95% CI −2 m 41 s to 1 m 5 s), p=0.37	114	96	18 (95% CI −53 to 17), p=0.3
Set A only	5 m 11 s	3 m 13 s	1 m 58 s (95% CI 0 m 36 s to 3 m 18 s), p=0.008	123	77	47 (95% CI 14 to 79), p=0.008
Set B only	5 m 38 s	4 m 53 s	0 m 44 s (95% CI −2 m 24 s to 0 m 54 s), p=0.35	107	93	14 (95% CI −45 to 18), p=0.37

**Figure 1:**
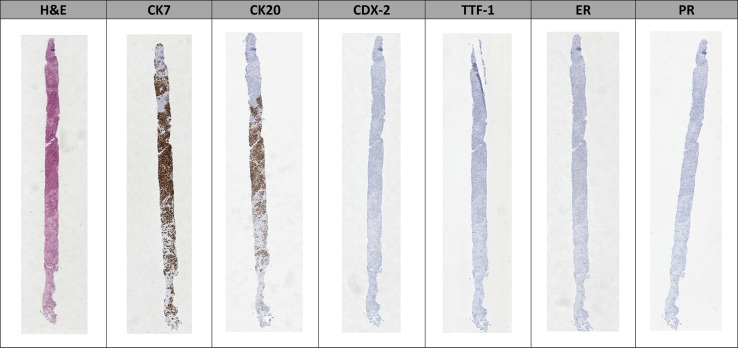
An example of a liver biopsy case used in this study. It includes a haematoxylin and eosin (H&E) stained slide alongside six other slides stained with typical immunohistochemical stains used in liver biopsy cases.

Each case contained one or more H&E-stained slides, as well as multiple slides stained with immunohistochemical stains that were relevant to the case. An example of the slides for a case can be seen in figure 1 below.

Before the experiment was undertaken, each participant was given a 15-minute training session using the digital microscope. A standard training session was divided into three sections: section one in which the researcher would show the participant how to use the software, section two where the participant would use the software themselves and the researcher would evaluate their use of it, and section three where they were asked to perform a diagnostic task.

All trials took place in a quiet, windowless room in the histopathology department of our institution usually used for teaching purposes. The digital microscope was placed at one end of the room with a light microscope set up on an adjacent table. The only light source was a standalone lamp placed next to the door behind the participant, to standardise the effect of ambient lighting on the display as far as possible at 10 lux.

**Figure 2:**
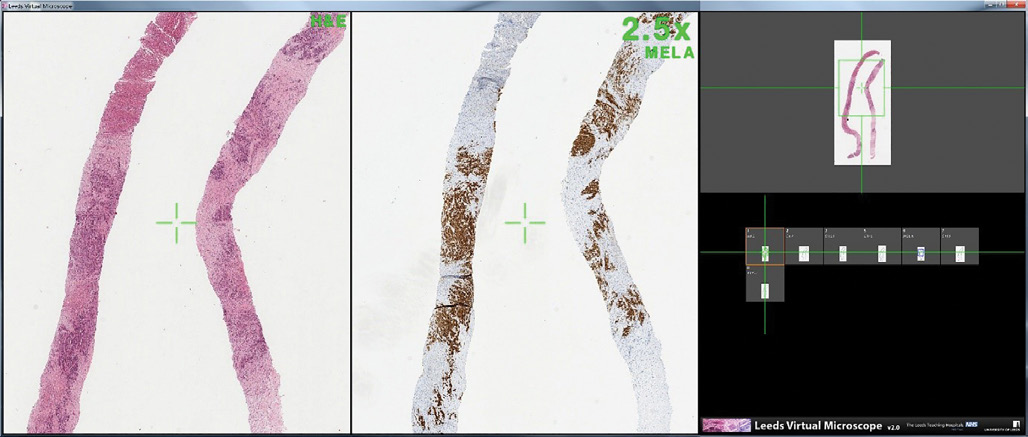
The screen layout of the digital microscope. The whole slide images are viewed on a large 6-megapixel medical grade display, and the thumbnails (right) are viewed on a smaller portrait 2-megapixel display to navigate between slides. The leftmost panel shows a high resolution (3 MP) view of the H&E-stained slide; the middle panel displays the currently selected immunostain. The two panels are synchronised so panning or zooming in one panel is replicated in the other. The user presses a key (space bar) to advance the next slide in the immunostain panel while the H&E image persists in the left panel.

A Dell Precision T5500 with AMD W5000 graphics card was used for this experiment. A Barco Coronis Fusion (6 MP) display (Barco Limited, Kortrijk, Belgium) was used in conjunction with a Barco Nio (2 MP) display (Barco Limited, Kortrijk, Belgium). The larger 30-inch screen was a split screen setup, the left side displaying the H&E slide (which was in constant display) and the right displaying the immunohistochemical slide. The smaller screen 21-inch on the right side of the participant displayed thumbnail images of all slides and highlighted which slide was currently in view. Viewing software was the Leeds Virtual Microscope.^11–13^ All digital slides were scanned on an Aperio T3 scanner (Leica Biosystems UK Limited, Milton Keynes, UK) with a ×40 objective lens at 0.25 μm per pixel. Images were compressed with conventional JPEG compression. Participants were able to pan a slide using a click and drag method. The keyboard was used to zoom and change slide. A screenshot of the digital microscope display can be seen in figure 2.

The light microscope was a Leica DMR microscope (Leica Biosystems UK Limited, Milton Keynes, UK) with ×2.5, ×5, ×10, ×20, ×40 and ×100 objectives and ×10 eyepiece. The microscope lens was reset by the researcher to the lowest magnification at the start of each trial (×2.5 magnification). The participants were provided with a practice slide in order to familiarise themselves with the microscope prior to the experiment.

The experiment was recorded using a three-video camera setup: one captured a ‘down-the-microscope’ view from the microscope camera mount, one captured the microscope work area (placed in the corner of the room furthest from the microscope) and the other directly captured the participants’ face and body from in front while at the digital workstation (placed directly behind the digital pathology workstation). Timing began when they picked up the first glass slide and finished when the set was completed. The video data were analysed to capture each instance of the participant interacting with the slide, panning, zooming, using the microscope condenser and writing notes. A screenshot of the participant video recording with the synchronised view down the microscope can be found in online supplemental figure 1.

10.1136/jclinpath-2021-207961.supp1 Supplementary data

Statistical analysis was performed in Stata V.16. Data approximated a normal distribution and therefore are summarised by the mean and SD.

As analyses indicated a wide variation in time to diagnosis according to the case, a normalised time to diagnosis (mean time to diagnosis for a case was calculated across both interfaces and then individual time to diagnosis on each interface expressed as a percentage of this time) is reported as the primary outcome measure. We also report actual time to diagnosis for ease of understanding.

Multiple linear regression was used to estimate the time to diagnosis in minutes adjusting for the binary fixed effects of experience level (trainee vs consultant) and interface (light vs digital microscope). CIs were generated to the 95% level. A sensitivity analysis of variance (ANOVA) was also performed on normalised time with the within-subject factor being light microscope versus digital microscope and between-subject variable being experience level (trainee vs consultant).

## Results

A summary of the main results can be seen in table 1; the normalised time results mirrored those of actual time across all outcomes.

**Figure 3:**
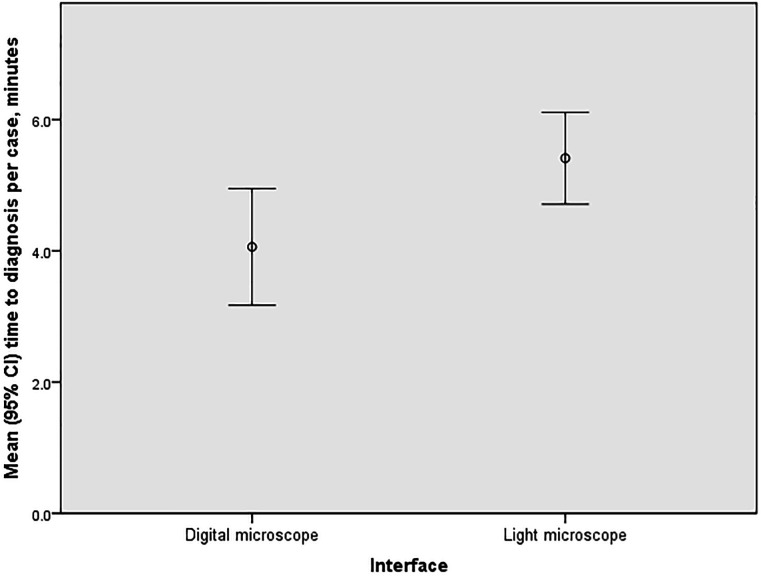
Mean time to diagnosis per case by interface. There was a statistically significant time-saving by using the digital microscope of 1 min 21 s (95% CI 16 s to 2 min 26 s; p=0.02) (bootstrapped p=0.009).

In terms of overall results, the mean time to diagnosis was 4 min 3 s using the digital microscope and 5 min 24 s using the light microscope, as shown in figure 3. This equates to a time-saving using the digital microscope of 1 min 21 s (95% CI 16 s to 2 min 26 s; p=0.02) (bootstrapped p=0.009).

**Figure 4:**
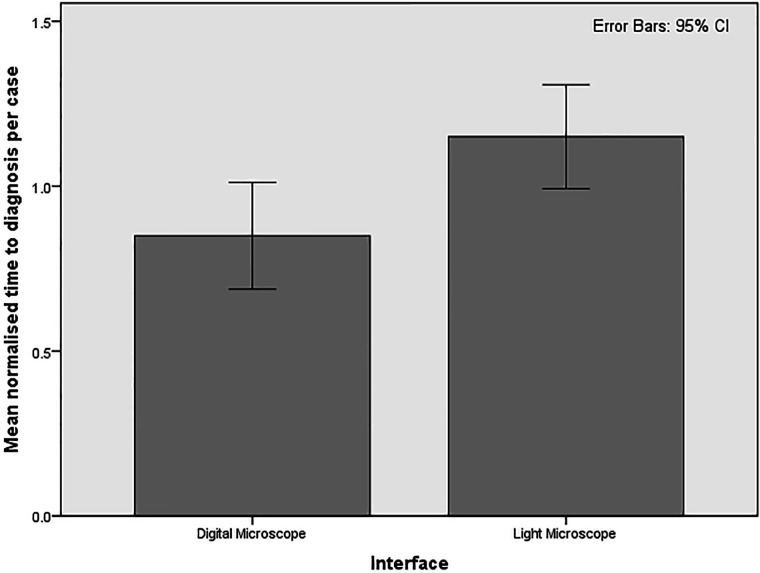
Percentage mean normalised time to diagnosis per case, with error bars showing 95% CIs. Overall normalised mean time to diagnosis was 85% on the digital pathology workstation compared with 115% on the microscope, a relative reduction of 26%.

Overall, normalised mean time to diagnosis was 85% on the digital pathology workstation compared with 115% on the microscope; a relative reduction of 26% (95% CI 15% to 45%; p=0.0006), as can be seen in figure 4.

When subcategorising the results by experience, the mean time to diagnosis for trainees using the digital microscope was 3 min 31 s, and 5 min 25 s using the light microscope. This equates to a time-saving using the digital microscope of 1 min 54 s (95% CI −3 min 11 s to −0 min 37 s; p=0.007). The mean time to diagnosis for consultants using the digital microscope was 4 min 35 s, as compared with 5 min 24 s using the light microscope. This results in a non-statistically significant time-saving using the digital microscope of 0 min 48 s (95% CI −2 min 41 s to 1 min 5 s; p=0.37). The normalised mean time to diagnosis for trainees was 74% and 116% compared with consultant times of 96% on the digital microscope and 114% on the light microscope, respectively. This equates to a reduction of 42% (95% CI 14% to 70%; p=0.006) for trainees, and again a non-statistically significant reduction of 18% (95% CI −53% to 17%; p=0.3) for consultants.

When evaluating the results by set, the mean time to diagnosis for set A across all participants using the digital microscope was 3 min 13 s, and 5 min 11 s using the light microscope. This equates to a time-saving using the digital microscope of 1 min 58 s (95% CI −3 min 18 s to −0 min 36 s; p=0.008). The mean time to diagnosis for set B across all participants using the digital microscope was 4 min 53 s, and 5 min 38 s using the light microscope, a difference which was not statistically significant (95% CI −2 min 24 s to 0 min 54 s; p=0.35). The mean normalised time to diagnosis for set A was 77% compared with 123%, set B 93% compared with 107% on the digital microscope and the light microscope, respectively. This equates to a statistically significant reduction in normalised time to diagnosis per case for set A of 47% (95% CI 14% to 79%; p=0.008), but again a non-statistically significant difference of 14% (95% CI −45% to 18%; p=0.37).

When combining the data across both interfaces, the data are summarised in table 2.

**Table 2:** The mean time to diagnosis across both interfaces by experience level and set

Mean time to diagnosis/case across both interfaces, (m=minutes; s=seconds)					
**Trainees only**	**Consultants only**	**Difference**	**Set A only**	**Set B only**	**Difference**
4 m 29 s	5 m 0 s	0 m 31 s (95% CI –1 m 42 s to 0 m 40 s), p=0.4	4 m 13 s	5 m 16 s	1 m 6 s (95% CI –2 m 11 s to 0 m 4 s), p=0.07

The mean time to diagnosis by trainees was 4 min 29 s, as compared with consultants with a mean time of 5 min 0 s. Therefore, trainees were faster across both modalities by a mean time of 31 s, but this difference was not significant (95% CI –1 min 42 s to 0 min 40 s; p=0.4). Similarly, when combining data across both interfaces, the mean time to diagnosis for set A cases was 4 min 13 s, and set B was 5 min 16 s, which represents a non-significant mean difference of 1 min 6 s (95% CI –2 min 11 s to 0 min 4 s; p=0.07).

Results of multivariable linear regression adjusting for experience level were largely the same as the unadjusted results presented above. Time to diagnosis on the digital microscope was 1 min 21 s faster than on the light microscope (95% CI 0 min 16 s to 2 min 26 s; p=0.017). Consultants took 31 s longer to reach diagnoses than trainees, but this was not statistically significant (95% CI –0 min 34 s to 1 min 36 s; p=0.34). There was an adjusted reduction in normalised time to diagnosis of 30% on the digital microscope as compared with the light microscope (95% CI –52% to −8%; p=0.008). There was an adjusted reduction in normalised time to diagnosis by trainees of 10% as compared with consultants but this was, again, non-significant (95% CI −32% to 11%; p=0.34). Similarly, there were no notable differences in the results of the sensitivity ANOVA performed on actual and normalised time, the details of which can be found in online supplemental table 2.

10.1136/jclinpath-2021-207961.supp3 Supplementary data

There were no major diagnostic errors made on either interface. Two participants gave a discordant diagnosis of hepatocellular carcinoma in a case of probable metastatic carcinoma—in set B. Review of the case revealed some cytological features that might support such a diagnosis and further immunohistochemistry (IHC) might be required to entirely rule it out, making the true diagnosis ambiguous. Therefore, either diagnosis was acceptable for the purposes of this study.

## Discussion

The significant difference in time to diagnosis demonstrates that for this set of diagnostic tasks, the digital microscope was quicker with a time-saving of 1 min 21 s per case, or a 26% relative reduction in time to diagnosis. As far as we are aware, this is the first study evaluating the impact of the digital microscope on time to diagnosis for cases involving immunohistochemically stained slides.

A mean reduction of 1 min 21 s per case using the digital microscope is a considerable time-saving in a health service that is struggling under ever-increasing demand for services. Previous work from our group has estimated the number of cases in our institution requiring extra stains to be approximately 5%. Given that our institution handles approximately 60 000 surgical cases per year, the use of the digital microscope for these cases alone may result in a time-saving of 67.5 histopathologist hours over the course of a year within our institution. This saving is likely to be higher in institutions where immunohistochemical or special stains are used more frequently.

The effect of increased experience of many of the specialist trainee participants with digital system was possibly reflected in the results, with the trainee cohort being just over 1 min quicker than the consultants on the digital pathology workstation per case, but this difference was not statistically significant (−1 min 4 s, 95% CI −2 min 49 s to 0 min 41 s; p=0.21). Alternatively, this may reflect the fact that trainee histopathologists were more familiar with liver specimens, as no consultant liver histopathologists were included in this study. However, no major diagnostic errors were made, thus suggesting unfamiliarity had no overall impact. Moreover, the use of only liver biopsies across the two interfaces will have prevented unfamiliarity with the type of case from biasing the results.

The time to diagnosis for set B was longer than the time to diagnosis for set A, although not statistically significant (mean difference of −1 min 6 s, 95% CI –2 min 11 s to 0 min and 4 s; p=0.07). This may have been due to some diagnostic difficulty surrounding one case; a diagnosis of hepatocellular carcinoma was made twice during the trial and given as a differential diagnosis once. If this was given only on the digital microscope, this may pose questions regarding image quality and the regulations regarding the use of digital slides for diagnostic work. However, these diagnoses were made on both interfaces: once on the digital microscope and the second on the light microscope. This would lead us to believe that these mistakes were due to the diagnostic difficulty surrounding of that case, as opposed to issues regarding image fidelity on the digital microscope.

Participants largely reported positive experiences using the digital microscope. A large proportion of participants used the system as intended and as was shown in the training session. However, some opted for a different technique, and rather than using the digital microscope at low power to identify an area of interest and then zoom, many participants used the digital microscope at very high magnification and scrolled the whole length of the image. This technique is inevitably time-consuming and may be due to unfamiliarity of the digital microscope for some participants; time reduction per case will likely increase with continued use and increased experience.

Fourteen participants reported that they found the two-screen digital microscope very useful for comparing areas of tissue side by side and aiding a diagnosis. Two participants commented on the controls and the ergonomics of the mouse and keyboard design and felt that a less cumbersome method of panning and switching between slides needed to be implemented. One participant commented that this implementation of a digital microscope to look at immunohistochemical cases was the best they had used to date. It is unsurprising that comments were not completely unanimous regarding the digital microscope workstation; it is well known that there is not a ‘one-size-fits-all’ workstation for digital radiologists.^14^

The design features of the digital microscope are a major strength to this study. The use of medical grade displays with high technical specifications enables more tissue to be viewed on the screen at one time reducing the need for interaction by the histopathologist (pan/zoom) for this low power assessment task; an 8-megapixel display shows approximately the same amount of tissue as a ×10 light microscope eyepiece. A high luminance and contrast ratio increases, the ability of the pathologist to make a confident diagnosis at low magnification. This, in combination with a viewer that facilities fast viewing, is likely responsible for the considerable time-saving afforded by the digital microscope in this study as this reduces refreshing time. Additionally, side-by-side viewing reduces cognitive load and streamlines the process of referencing the H&E WSI to check an area for relevance; light microscopy demands that pathologists remove the IHC glass slide, load the H&E glass slide and navigate to the area of interest, which is challenging and time-consuming.

Previous work from our group did not demonstrate a time-saving from increased screen resolution,^3^ but the discrepancy between those results and this study is likely due to three main factors. First, that work involved a very specific search task (identifying micrometastases), as opposed to a more general low power assessment in this work. Second, the high-resolution displays were split across three screens in the previous work; the bevels were a hinderance to pathologists as they had to ensure that the micrometastasis was not being obscured by the bevels. Third, this study involved updated viewing software that was faster to respond to the user.

Another study by Hanna *et al* 15 found that the digital microscope was 19% slower than using the light microscope. There are again many reasons why our results were not in agreement with their findings. First, they included relatively inexperienced users; a large-scale validation study by our group^16^ demonstrated that experience of between 2 and 6 months is required for users to become proficient. Second, they employed the use of small monitors (24”) with relatively low resolution (1920×1200), equating to just 2.3 MP. Third, they used a custom Graphical User Interface which can present many difficulties in the initial phases and evolve over time, as outlined during our development of the Leeds Virtual Microscope which was initiated back in 2007.^17^ A very recent study by Borowsky *et al* 18 found that the digital microscope took an average of 5.20 min as compared with 4.95 min on the light microscope. However, again, there are reasons for this discrepancy with our findings; this study included only 25% slides that were IHC or special stains and does not mention the digital microscope setup other than the use of Dell medical grade monitors. In our experience, many factors (user experience and training, details of the user interface design, task choice and technical display specifications) can all affect time to diagnosis. Objective comparison of these factors is difficult as there are complex interactions between them. Mills *et al* 19 found that when including a range of surgical specimens, digital diagnosis took 4 s longer than the light microscope, but as highlighted by the authors the slowest reader got considerably quicker with digital diagnoses over the course of the study, and was similar to the light microscope by the end of the study. This demonstrates nicely that all studies of this nature are biased in favour of the light microscope due to relative inexperience with their digital counterparts. It also highlights the need to include suitable training in future longer-term studies of efficiency or time to diagnosis.

The effect of working digitally does not just result in potential time-savings in time to diagnosis, but instead impacts the entire laboratory workflow. Although it is outside of the scope of this work to discuss the impact of a whole system evaluation of digitised pathology services, this has recently been addressed by the work of Baidoshvili *et al*.^20^ They focus on the time-savings across the entire pathology workflow when comparing analogue with digital rather than just the time to diagnosis and found that there were time-savings of approximately 19 hours within a working day across a pathology laboratory, equating to 2.63 full-time equivalent staff. Further work should be conducted prospectively on the cost:benefit as departments become fully digital.

Inevitably, there were several limitations to our work. First, this was a small study with known considerable user variation. Second, the time difference to diagnosis will be affected by the participant’s familiarity with the case type. Although this should not impact the primary outcome in this study (difference between the two interfaces), having participants diagnosing case types that they are familiar with may be more reflective of the time-savings observed in routine clinical practice. It should also be noted that many routine cases do not require immunohistochemical slides to review and therefore the observed time-saving may not be applicable to these cases. Third, it would be advantageous to spend longer familiarising participants with the digital microscope; familiarity with one interface and not the other will inevitably bias the results in favour of the familiar interface. Lastly, due to the prototype nature of the digital microscope, the usability could be improved. There were some issues regarding slide registration (alignment of the H&E and immunostain images), which proved particularly problematic for participants who were less adept with the digital microscope. Further, time-saving will be likely observed as the digital microscope becomes more user-friendly.

## Conclusions

To conclude, the digital microscope reduced time per case by 1 min 21 s per case and a relative reduction of 26%, without any major diagnostic errors as compared with the light microscope. This is likely due to the ability to view multiple slides simultaneously, which is not possible using analogue systems. We anticipate that these time-savings will have a major improvement on pathologist productivity at a time where pathology services are strained, and serve as a point from which to build other user interfaces to enhance pathologist productivity.

**Take home messages**

- Digital pathology may offer benefits over microscope in viewing whole slide images—one unique capability is the ability to review side by side, with synchronised pan and zoom.
- We designed a pragmatic study looking at evaluation of liver biopsy cases including an immunohistochemical panel, where serial comparisons are needed.
- We used a custom viewer that allowed side-by-side viewing, and rapid review of immunohistochemical images in the sequence.
- We found that mean time was 5 min 24 s on the light microscope and 4 min 3 s on digital microscope, a reduction of 1 min 21 s (95% CI 16 s to 2 min 26 s; p=0.02) and a relative reduction of 26%. These benefits were seen with relatively little training and exposure to the system, and further work is needed to evaluate the real-world impact.

## Data Availability

All data relevant to the study are included in the article or uploaded as supplemental information. To access the raw data for this study please contact the corresponding author.
