# Supplementary material for: Faster than light (microscopy): superiority of digital pathology over microscopy for assessment of immunohistochemistry
Source: J Clin Pathol. 2022 Jan 17;76(5):333–8. doi: 10.1136/jclinpath-2021-207961 (PMC10176378; doi:10.1136/jclinpath-2021-207961)
Supplement: Supplementary data [file jclinpath-2021-207961supp002.pdf]

Supplemental Table 1

Set	Case	Clinical Details	Number of slides	Immunohistochemical stains	Diagnosis
A	1	47-year-old female. Lung hilar mass. Multiple liver metastasis.	8	H&E, CD45, CD56, CHROMO, CK7, CK20, synaptophysin, TTF1	Small cell carcinoma
	2	68-year-old female. ?Gallbladder mass invading liver.	6	H&E, CK7, CK19, CK20, P63, TTF-1	Carcinoma with squamous features
	3	41-year-old male. Pancreatic mass. Multiple liver metastases.	7	H&E, CDX-2, CK7, CK20, ER, PR, TTF-1	Adenocarcinoma
B	1	58-year-old female. Liver metastases seen on CT. Previous breast carcinoma.	6	H&E, GCDFP-15, ER, PR, HER-2, HER-2	Carcinoma
	2	75-year-old male. ?Metastatic lung cancer	8	H&E, CAM 5.2, CD56, CK5, CK14, MNF, P63, TTF-1	Squamous cell carcinoma

	3	76-year-old female. Admission with pain, hepatomegaly and weight loss. ?Metastatic or primary liver malignancy.	7	H&E, CDX-2, CK7, CK20, ER, PR, TTF-1	Adenocarcinoma

***Supplemental Table 1** – Case details provided to the participants. Cases were chosen to include equal numbers of slides in each set. Please note the clinical details are fictitious in the interests of confidentiality.*
