# Supplementary material for: Faster than light (microscopy): superiority of digital pathology over microscopy for assessment of immunohistochemistry
Source: J Clin Pathol. 2022 Jan 17;76(5):333–8. doi: 10.1136/jclinpath-2021-207961 (PMC10176378; doi:10.1136/jclinpath-2021-207961)
Supplement: Supplementary data [file jclinpath-2021-207961supp001.pdf]

Supplementary Figure 1

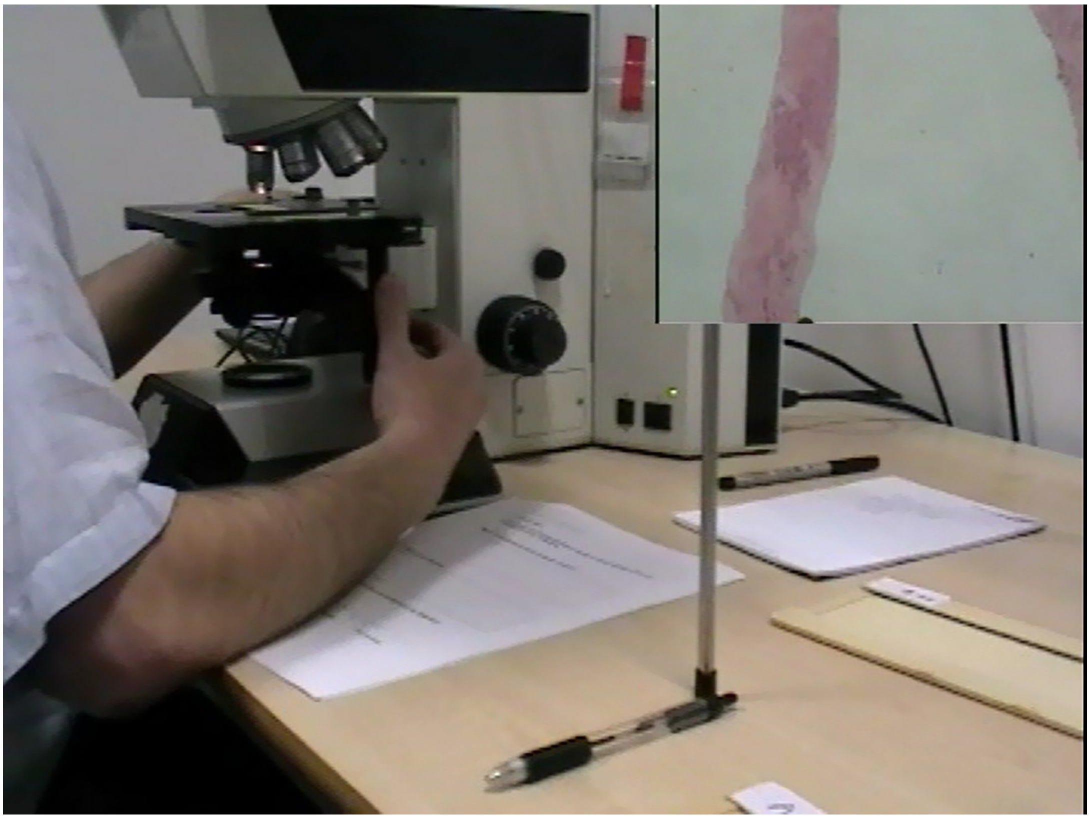

Supplementary Figure 1 *– Participant video recording with synchronised view down the microscope. Here a participant can be seen operating the light microscope with the answer sheet to their right. The view inset (top right) shows the view down the microscope as it was being recorded. Permission has been sought from the participant in the image.*
