# Supplementary material for: Faster than light (microscopy): superiority of digital pathology over microscopy for assessment of immunohistochemistry
Source: J Clin Pathol. 2022 Jan 17;76(5):333–8. doi: 10.1136/jclinpath-2021-207961 (PMC10176378; doi:10.1136/jclinpath-2021-207961)
Supplement: Supplementary data [file jclinpath-2021-207961supp003.pdf]

Supplemental Table 2

Number of obs =	32	R-squared =	0.2039
Root MSE =	1.5038	Adj R-squared =	0.1490

Source	Partial SS	df	MS	F	Prob>F
Model	16.794996	2	8.397498	3.71	0.0367
interface	14.624719	1	14.624719	6.47	0.0166
experience	2.1702771	1	2.1702771	0.96	0.3354
Residual	65.580912	29	2.2614108		
Total	82.375908	31	2.6572874		

Supplemental Table 2A - *Results of the sensitivity ANOVA on normalised time.*

Number of obs =	32	R-squared =	0.2365
Root MSE =	.30009	Adj R-squared =	0.1839

Source	Partial SS	df	MS	F	Prob>F
Model	.80909562	2	.40454781	4.49	0.0200
interface	.7236045	1	.7236045	8.04	0.0083
experience	.08549113	1	.08549113	0.95	0.3379
Residual	2.6115643	29	.09005394		
Total	3.4206599	31	.11034387		

Supplemental Table 2B - *Results of the sensitivity ANOVA on normalised time.*
